# Metabolic control of acclimation to nutrient deprivation dependent on polyphosphate synthesis

**DOI:** 10.1126/sciadv.abb5351

**Published:** 2020-09-30

**Authors:** E. Sanz-Luque, S. Saroussi, W. Huang, N. Akkawi, A. R. Grossman

**Affiliations:** 1 Department of Plant Biology, The Carnegie Institution for Science, 260 Panama Street, Stanford, CA 94305, USA.; 2 Department of Biology, Stanford University, Stanford, CA 94305, USA.

## Abstract

Polyphosphate, an energy-rich polymer conserved in all kingdoms of life, is integral to many cellular stress responses, including nutrient deprivation, and yet, the mechanisms that underlie its biological roles are not well understood. In this work, we elucidate the physiological function of this polymer in the acclimation of the model alga *Chlamydomonas reinhardtii* to nutrient deprivation. Our data reveal that polyphosphate synthesis is vital to control cellular adenosine 5′-triphosphate homeostasis and maintain both respiratory and photosynthetic electron transport upon sulfur deprivation. Using both genetic and pharmacological approaches, we show that electron flow in the energy-generating organelles is essential to induce and sustain acclimation to sulfur deprivation at the transcriptional level. These previously unidentified links among polyphosphate synthesis, photosynthetic and respiratory electron flow, and the acclimation of cells to nutrient deprivation could unveil the mechanism by which polyphosphate helps organisms cope with a myriad of stress conditions in a fluctuating environment.

## Teaser

Acclimation to nutrient limitation requires polyP synthesis to control ATP homeostasis and sustain respiration and photosynthesis.

## INTRODUCTION

Polyphosphate (polyP), an ancient polymer conserved in organisms from bacteria to humans, is composed of phosphate groups linked by the highly energetic phosphoanhydride bond found in adenosine 5′-triphosphate (ATP). PolyP is abundant in cells, with concentrations that range from micromolar in mammals to millimolar in unicellular prokaryotes and eukaryotes (*1*). This polymer mainly accumulates in acidocalcisomes, acidic vacuoles that store calcium and other divalent cations in both eukaryotes and prokaryotes (*2*), although polyP can also localize to the cell wall, mitochondria, nucleus, and endoplasmic reticulum (*3*). In bacteria and amoebae such as *Dictyostelium discoideum*, polyP synthesis is mediated by polyP kinases (*4*–*6*), whereas in most unicellular eukaryotes, the synthesis is performed by the vacuolar transporter chaperone (VTC) complex (*7*). In both cases, the terminal phosphate of ATP is transferred to the growing polyP chain, resulting in the accumulation of energy in orthophosphate bonds and the release of adenosine 5′-diphosphate (ADP). In higher eukaryotes, although polyP and acidocalcisomes are present (*2*, *8*), the mechanism for polyP synthesis is still unknown.

In yeast, the VTC complex contains three polypeptide components, VTC1, VTC4, and VTC2/VTC3 (*9*). VTC4, the catalytic subunit of the complex, has three functional domains. The N-terminal region contains the SPX domain, which binds inositol pyrophosphate and activates polyP polymerase activity, the central region includes the catalytic polyP polymerase domain, and the C-terminal region contains the VTC domain, which consists of three α helices that anchor the protein to the membrane (*7*, *10*–*12*).

Intracellular polyP aggregates were found more than a hundred years ago, and over the past three decades, many studies have shown that different organisms require polyP to cope with various stress conditions including high temperature, oxidative stress, osmotic shock, survival during stationary phase, heavy metal accumulation, and nutrient deprivation, among others (*1*, *5*, *13*–*18*). Moreover, polyP synthesis affects critical cellular processes, including DNA replication, transcription, translation, quorum sensing, and protein folding, and is essential for normal infectivity of many parasites (*1*, *5*, *18*–*23*). However, how this simple polymer can be involved in so many different processes is an unanswered and intriguing question.

Researchers are just beginning to unravel mechanisms by which polyP regulates a broad spectrum of biological processes. A recently found posttranslational modification, polyphosphorylation, involves the covalent attachment of polyP to lysine residues. This modification is present on proteins involved in ribosome biogenesis and DNA unfolding (topoisomerases) (*24*, *25*). Another feature of polyP that could explain its impact on numerous cellular processes is its ability to serve as a stabilizing scaffold for protein-folding intermediates and for the protection and maintenance of protein activities by preventing their aggregation, as reported for bacterial cells exposed to high temperature or oxidative stress (*13*, *26*). Similarly, low levels of polyP have been associated with protein aggregation (amyloid plaques) in neurons and neurodegenerative diseases (*13*). PolyP can also interact with proteases and promote the degradation of specific proteins. For example, the Lon protease interacts with polyP, activating degradation of ribosomal polypeptides during amino acid starvation of *Escherichia coli* (*21*). Last, polyP can serve as a chelator of divalent cations (Ca^2+^, Fe^2+^, Cd^2+^, Mn^2+^, and Cu^2+^, among others) and control intracellular cation availability (*15*, *27*). Despite this growing number of discoveries concerning the impact of polyP on cellular processes, many with seemingly unrelated functions, we still know little about how this polymer and its dynamic synthesis and degradation help cells acclimate to stress.

To elucidate the link between polyP and stress acclimation, we focused on nutrient deprivation. Organisms starved for sulfur (S) trigger a suite of responses that include induction of genes encoding high-affinity transporters and assimilatory enzymes responsible for efficient scavenging and utilization of the limiting nutrients, the reorganization of cellular metabolism and energetics (*28*, *29*), and the accumulation of polyP (*16*, *30*). In the green alga *Chlamydomonas reinhardtii* (*Chlamydomonas* throughout), the VTC1 protein, which only contains the VTC domain and seems to have a structural function in the VTC complex, is essential for polyP accumulation. Strains lacking this protein were unable to normally acclimate to S deprivation (*16*). However, the role of polyP in the acclimation of cells to nutrient deprivation is still not clear.

Here, we show that, similar to the phenotype of the *vtc1* mutant (*16*), the loss of VTC4 in *Chlamydomonas* leads to impaired acclimation to sulfur deprivation, but we also elucidate the connection between polyP and the acclimation process, demonstrating that polyP-deficient strains initiate the acclimation process but are unable to sustain it. *Chlamydomonas* cells appear to need polyP synthesis to maintain the ATP homeostasis and levels of adenylates in cells, which is critical to avoid inhibition of electron flow in both mitochondria and chloroplasts. Moreover, we demonstrate that active electron flow, independent of the cellular ATP level, is necessary to induce the responses associated with S and N deprivation. We suggest that the links between the synthesis of polyP, maintenance of intracellular ATP levels, sustained chloroplast and mitochondrial electron flow, and the specific responses of the cells to stress represent a set of interacting activities that is conserved in many organisms and that becomes critical under conditions in which the cells are able to synthesize high levels of ATP but are unable to grow. This work integrates key roles of polyP synthesis in regulating the energetics of the cell and provides insights into how polyP can affect numerous cellular processes, including the ways in which the cells cope with changing environmental conditions.

## RESULTS

### VTC4 is essential for “sustaining” the acclimation to S deprivation

**Fig. 1:**
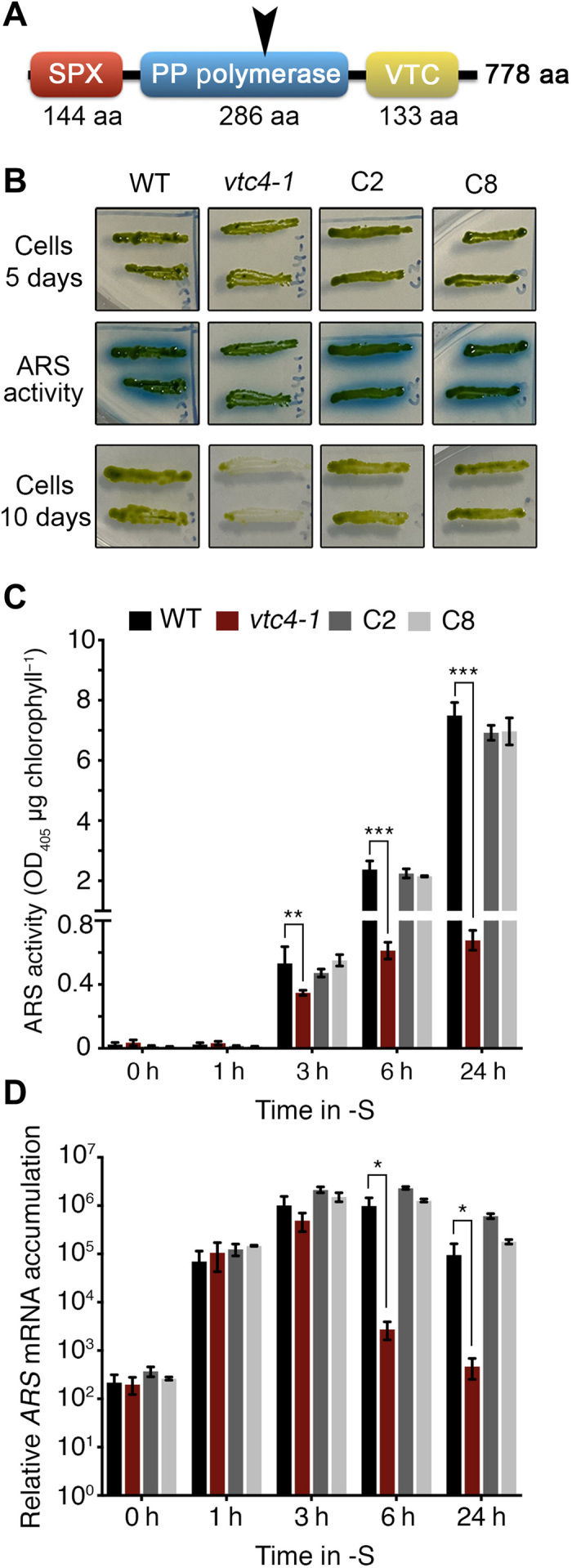
VTC4 is required to sustain acclimation to sulfur deprivation. (**A**) Conserved domains of VTC4 and the position at which the protein is truncated in the *vtc4-1* mutant (black arrowhead). aa, amino acid; PP, polyP. (**B**) ARS activity (blue signal) in TAP-S solid agar medium. TAP-grown cells were induced on TAP-S agar medium for 5 and 10 days. ARS activity was measured with cells incubated for 5 days in TAP-S. Photo credit: Emanuel Sanz-Luque, Carnegie Institution for Science. (**C**) Quantified ARS activity from liquid medium and (**D**) *ARS* mRNA accumulation following TAP-S induction. Error bars represent ±SD, *n* ≥ 3. Student’s *t* test was performed. *P < 0.05, **P < 0.01, and ***P < 0.001. OD_450_, optical density at 450 nm.

The VTC4 protein is highly conserved, and as in other organisms, the *Chlamydomonas* protein contains three domains (SPX, polyP polymerase, and VTC) that were previously described in yeast and trypanosomatids (*7*, *10*, *31*) (Fig. 1A and fig. S1). To elucidate the function of polyP in acclimation to S deprivation, we characterized two mutants disrupted for the gene encoding VTC4, which has not been previously characterized in algae. These mutants were obtained from a genome-wide mutant library generated by insertional mutagenesis (*32*). In the *vtc4-1* mutant, the insertion was in the middle of the polyP polymerase domain, without causing a deletion or rearrangement at the insertion site (Fig. 1A and fig. S2). In the second allelic mutant (*vtc4-2*), the insertion occurred just downstream of the polyP polymerase domain and caused a deletion of 4120 base pairs (bp; fig. S3, A and B).

To assess the impact of the loss of VTC4 on S deprivation responses (SdR), we quantified the activity of arylsulfatase (ARS), a sulfur deprivation–induced periplasmic enzyme involved in scavenging sulfate from organic molecules in the environment. This enzyme has been extensively used as a reporter of the SdR (*33*). The two allelic *vtc4* mutants showed very low ARS activity following transfer to medium depleted for S [tris acetate phosphate (TAP)–S], while the ectopic expression of a wild-type (WT) *VTC4* gene in the *vtc4-1* mutant (C2- and C8-complemented lines) rescued the ARS phenotype (Fig. 1B and fig. S3C). Moreover, the *vtc4-1* mutant, but neither WT cells nor the complemented lines, bleached after 10 days of S deprivation (Fig. 1B), which confirmed the essential role of VTC4 in the acclimation process.

In a previous study, it was shown that the *vtc1* mutant exhibited low *ARS* mRNA accumulation after 6 hours of S deprivation (*16*). To acquire a better understanding of how the lack of the VTC complex affects the acclimation process, we studied the kinetics of ARS activity and mRNA accumulation in S-depleted medium. Unexpectedly, ARS activity and mRNA accumulation (Fig. 1, C and D) increased similarly in WT and *vtc4-1* for the first ~3 hours of starvation. However, the *ARS* mRNA level in the mutant dropped markedly by 6 hours of S deprivation and reached preinduction levels after 24 hours (Fig. 1D). The ARS enzyme, on the other hand, is very stable, and its activity was identical after 6 and 24 hours of S deprivation (Fig. 1C). These results clearly indicate that VTC4 is not necessary to trigger acclimation but is required to sustain the SdR (at least with respect to *ARS* expression). The initial induction of *ARS* followed by a “repressor effect” (Fig. 1, C and D) in the *vtc4-1* mutant was not exclusive for *ARS* mRNA but was also observed for other transcripts induced during S deprivation. The transcripts for two high-affinity sulfate transporters [sulfate transporter 2 (SULTR2) and SAC1-like transporter 1 (SLT1)] and a cell wall protein with almost no S-containing amino acids [extracellular protein ~76 kDa (ECP76)] (*28*, *29*) were also quantified (fig. S4, A to C). The genes encoding these proteins belong to the two different tiers of regulation identified for SdR. Genes in the first tier encode mainly transporters (for S scavenging), which are induced immediately after deprivation. The second-tier genes are induced later in the acclimation process and are associated with sustained deprivation conditions (*ARSs* and *ECPs*, among others) (*34*). All of these transcripts were similarly induced after 1 to 3 hours of S limitation in WT, *vtc4-1*, and the C2- and C8-complemented strains, while, at later times, only the mutant exhibited a reduction of more than 10-, 5-, and 56-fold for *SULTR2*, *SLT1*, and *ECP76*, respectively (fig. S4, A to C). Our results indicate that VTC4 has a broad impact on sustaining the transcriptional activity of the SdR genes, but the final transcript levels may vary depending on which tier of control they are associated with and the stability of their transcripts under deprivation conditions.

To test whether the observed results reflect a general effect on transcription, we quantified the ACT1 (actin) and GPX5 (glutathione peroxidase) transcripts. The levels of these transcripts were not affected in the S-deprived *vtc4-1* mutant (fig. S4, D and E). These results indicate that the lack of or inability to synthesize polyP does not affect transcription in general and appears to be specific to mRNAs associated with the SdR.

We also observed that the inability of the *vtc4-1* mutant to acclimate to S deprivation resulted in a slight effect on growth after S replenishment. The S-depleted mutant cells exhibited a ~50% reduction in growth over a period of 24 hours after being transferred to nutrient-replete medium. By 48 hours following the transfer, as the cells approached stationary phase, the cells showed approximately the same level of growth as the WT and complemented strain (fig. S5). These results indicate that after being starved for S, the mutant initially may have a slower metabolism and/or reduced capacity to assimilate S compared to the WT and the complemented strain (the WT and complemented strain would have higher levels of transcripts for the high-affinity S transporters and potentially other acclimation-associated proteins following growth in S-depleted medium).

### VTC4-dependent polyP synthesis in both S-replete and S-depleted media

**Fig. 2:**
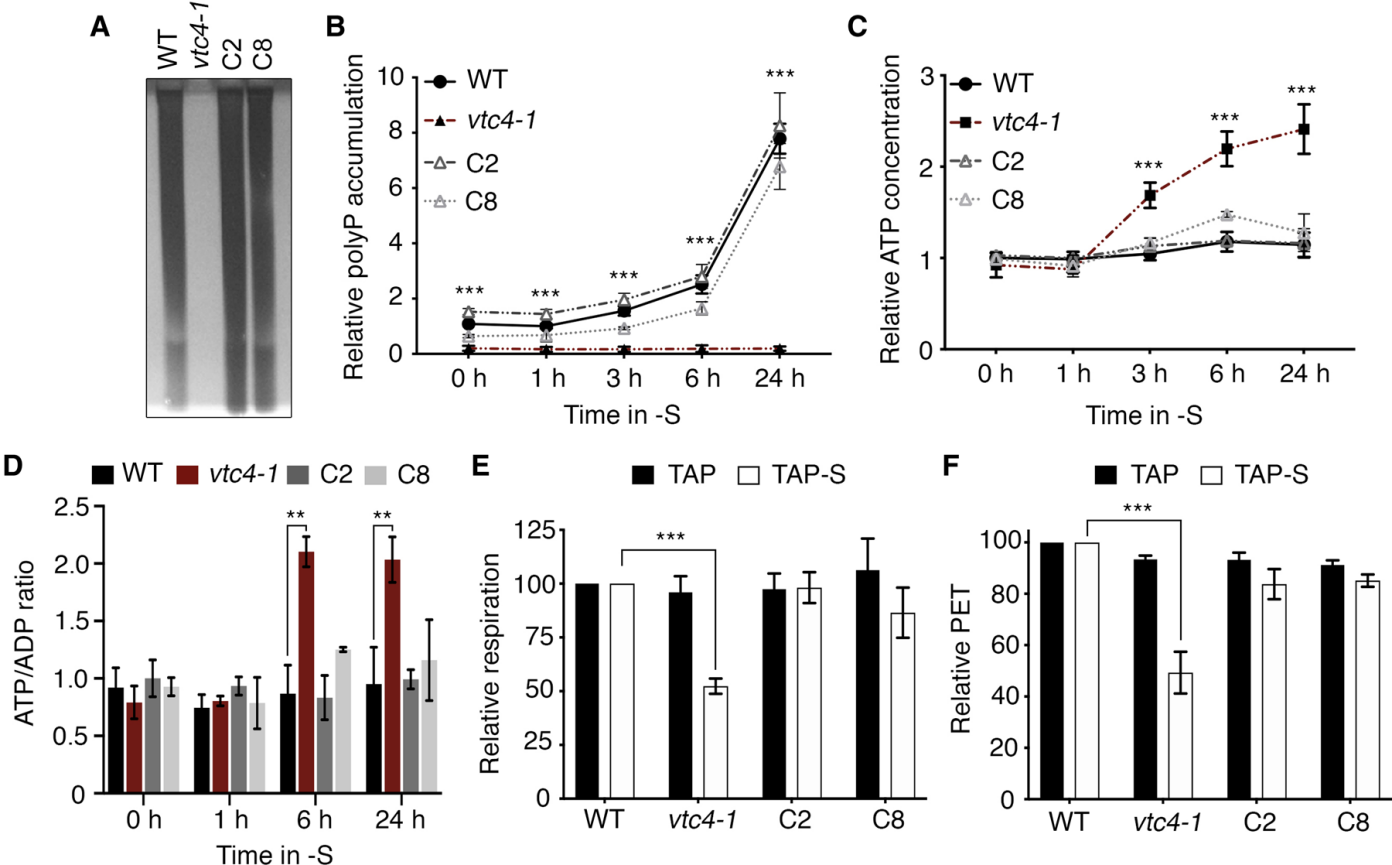
Lack of polyP synthesis leads to ATP accumulation and reduces the rate of respiratory and photosynthetic electron transport. (**A**) PolyP detected by polyacrylamide gel electrophoresis analysis in nutrient-replete conditions. (**B**) Relative polyP levels before (time, 0 hours) and after induction in TAP-S. Levels of polyP were normalized to that of WT cells in nutrient-replete conditions. (**C**) Relative intracellular ATP concentration and (**D**) ATP/ADP ratio under nutrient-replete conditions (time, 0 hours) and as cells become S deprived. (**E**) Relative respiration and (**F**) photosynthetic electron transport (PET) rates in nutrient-replete conditions (TAP) and after 6 hours of S deprivation (TAP-S) in cultures at 10 μg/ml chlorophyll. Error bars represent ±SD, *n* ≥ 3. Student’s *t* test was performed. **P < 0.01 and ***P < 0.001. PolyP and ATP concentrations that correspond to WT at time 0 hours (value of 1 in the graph) were 265.4 ± 44.22 μmol polyP μg chlorophyll^−1^ and 92.7 ± 2.02 pmol ATP μg chlorophyll^−1^, respectively. For WT cells, respiration values were 40.6 ± 2.7 and 38.8 ± 3.2 μmol O_2_ mg chlorophyll^−1^ hour^−1^ in TAP and TAP-S, respectively. PET values for WT were 26.2 ± 0.3 in TAP and 20.4 ± 0.2 μmol photons m^−2^ s^−1^ in TAP-S. WT values were considered as 100%, and the values for the other strains under the same conditions were normalized to the WT values.

The accumulation of polyP under nutrient stress conditions has been previously reported in different organisms, including *Chlamydomonas*, although we know little about the kinetics of accumulation. To examine the correlation between the development of the “*ars*” phenotype and polyP accumulation, we quantified polyP in cells maintained in nutrient-replete conditions and those exposed to 1, 3, 6, and 24 hours of S deprivation. *Chlamydomonas* WT cells accumulated low levels of polyP when grown in mixotrophic, nutrient-replete conditions (TAP). In contrast, no polyP was detected in either the *vtc4-1* or *vtc4-2* mutants; the phenotype was rescued in the complemented lines (Fig. 2A and fig. S6). Quantification of polyP in WT cells and the complemented *vtc4-1* strains demonstrated an ~8-fold increase after 24 hours of S deprivation, with no polyP detected in the *vtc4-1* mutant under the same conditions (Fig. 2B and fig. S6, B and C). PolyP accumulation in WT and the complemented lines was not observed immediately but only after ~3 hours of S deprivation, which correlated with the time at which we observed transcriptional repression of the SdR. These results clearly establish that the initial pool of polyP is not required for the induction of the SdR. This finding raises the possibility that an inability of the mutant to synthesize polyP following 3 hours of S deprivation might be the cause of its “*ars^−^*” phenotype.

### Lack of polyP synthesis leads to high ATP accumulation and reduced electron flow in chloroplasts and mitochondria

PolyP synthesis involves ATP hydrolysis and the regeneration of ADP (*7*). Therefore, we hypothesized that this polymer could serve as an energy buffer to reduce the concentration of excess ATP during S deprivation when growth, the primary sink for energy, is arrested. We measured ATP levels in WT, *vtc4-1*, C2, and C8 strains grown in both replete medium and in medium lacking S. While a loss of VTC4 did not affect ATP levels in the cells during the first hour of S deprivation, after 3 hours, the cellular ATP concentration of *vtc4-1* rose, with a ~2.5-fold increase after 24 hours (Fig. 2C). This accumulation in the mutant paralleled a rise in the ATP/ADP ratio (Fig. 2D). In contrast, the intracellular ATP concentrations for the WT and complemented strains were stable at all time points after exposing the cells to S deprivation. The kinetics of ATP accumulation in the mutant was similar to that of polyP accumulation in WT cells (compare Fig. 2, B and C), suggesting a connection between polyP synthesis and ATP homeostasis and a previously unrealized potential link between cellular energetics and maintenance of the SdR.

A high intracellular ATP/ADP ratio is known to inhibit ATP synthesis, which is coupled to electron transport and the dissipation of a proton gradient across the thylakoid and respiratory membranes (*35*, *36*). To assess the impact of high ATP on respiration in the mutant, we quantify mitochondrial O_2_ uptake (respiration) under nutrient-replete and S-deprivation conditions. The WT and *vtc4-1* strains exhibited almost the same respiration rate under nutrient-replete conditions, but *vtc4-1* showed a ~50% reduction relative to WT when the cells were deprived of S for 6 hours (Fig. 2E). The mutant cells were similarly inhibited for photosynthetic electron transport (PET; Fig. 2F). Dark-acclimated WT and *vtc4*-*1* mutant cells showed a similar maximum photosystem II (PSII) yield (Fv/Fm) after 6 hours of S deprivation (fig. S7A), which indicates that PSII is not damaged in the mutant. However, after illumination, the PSII yield in the mutant displayed a more pronounced drop than in WT cells (fig. S7B); this reduction is congruent with a restriction in photosynthetic electron transport (PET) caused by a high intracellular ATP concentration that is generated in strains with no polyP polymerase activity. These data also suggest the importance of polyP synthesis in maintaining ATP homeostasis, which, in turn, is required to sustain electron flow in both mitochondria and chloroplasts during acclimation to S deprivation.

### Reduction of ATP levels allows rescue of the *vtc4-1* mutant phenotype

**Fig. 3:**
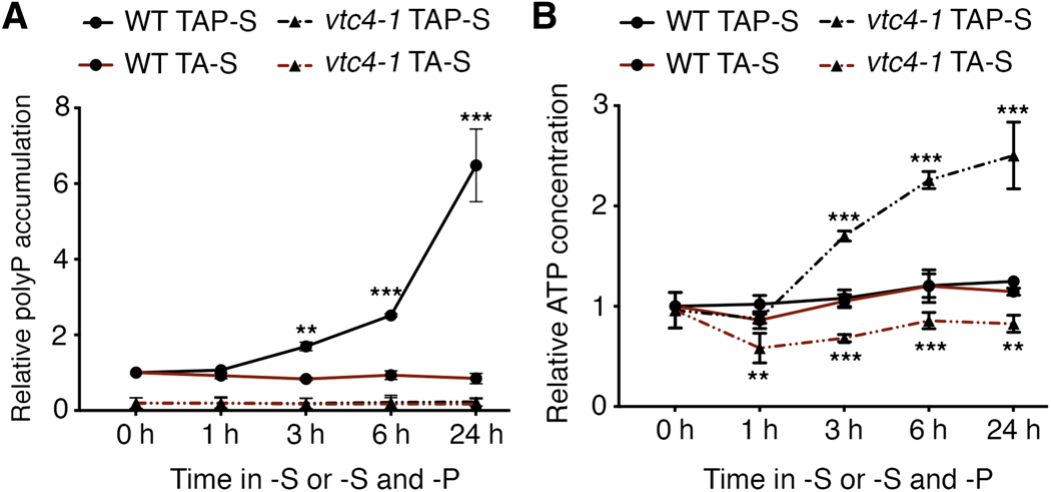
Accumulation of polyP and ATP in WT and *vtc4-1* deprived of S and of S and P. (**A**) Relative polyP accumulation and (**B**) ATP concentration after S deprivation (TAP-S) or both S and P deprivation (TA-S) in WT and the *vtc4-1* mutant. PolyP and ATP values for the WT strain at time 0 hours (value of 1 in the graph) were 488.66 ± 76.95 μmol polyP μg chlorophyll^−1^ and 82.96 ± 8.84 pmol ATP μg chlorophyll^−1^, respectively. Error bars represent ±SD, *n* ≥ 3. Student’s *t* test was performed. **P < 0.01 and ***P < 0.001.

**Fig. 4:**
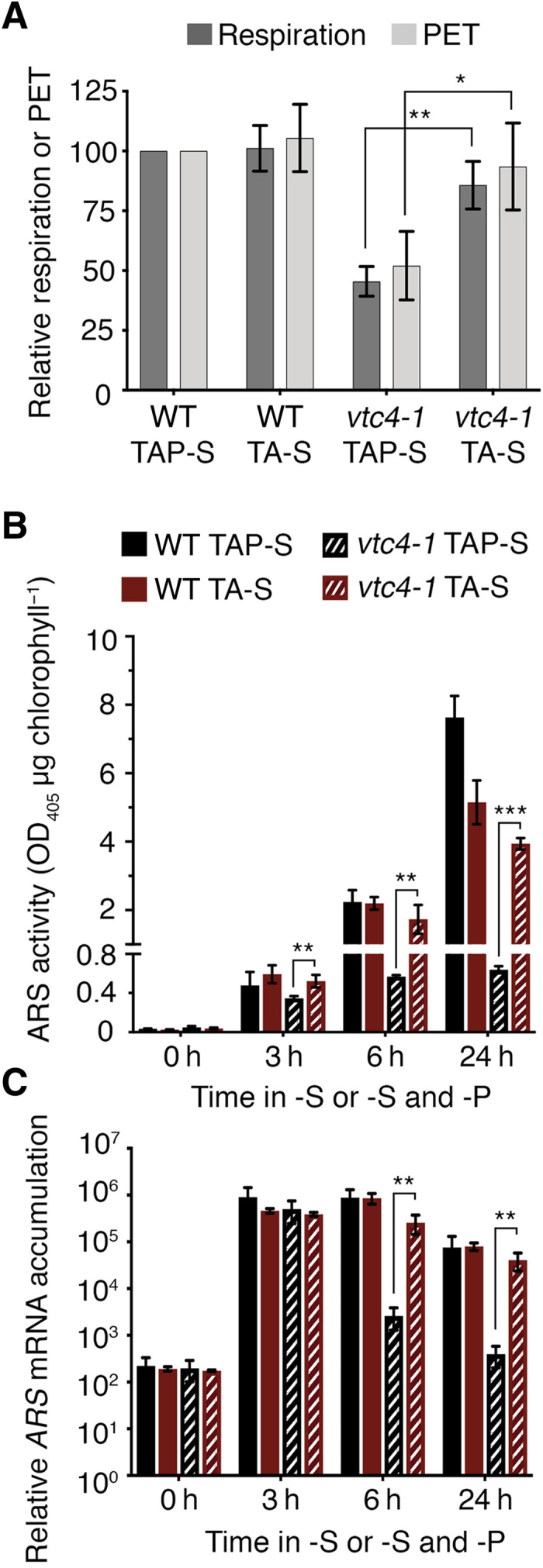
PolyP synthesis is not required for the acclimation to S deprivation in the absence of P. (**A**) Relative respiration and PET for WT and *vtc4-1* after 6 hours in TAP-S medium or in medium depleted of both S and P (TA-S). Cultures at 10 μg/ml were used to measure O_2_ uptake and PET, and WT values in TAP-S were considered as 100% and used for the normalization. A value of 100% corresponds to 48.6 ± 6.0 μmol O_2_ mg chlorophyll^−1^ hour^−1^ for respiration and 20.3 ± 2.1 μmol photons m^−2^ s^−1^ for PET. (**B**) ARS activity and (**C**) relative *ARS* mRNA levels after -S and -S plus -P deprivation (TA-S). Error bars represent ±SD, *n* ≥ 3. Student’s *t* test was performed. *P < 0.05, **P < 0.01, and ***P < 0.001.

To determine whether the phenotypes of *vtc4-1* (abnormal acclimation and reduced respiration/photosynthesis) were a consequence of the high ATP levels or impaired polyP synthesis, we deprived mutant cells of S under conditions in which, in addition to not making polyP, the cells were unable to accumulate ATP. This situation was created by placing cells in medium depleted of both S and phosphorus (P; TA-S medium); the S-deprived mutant in the absence of Pi still did not accumulate polyP but was now also unable to accumulate ATP (Fig. 3, A and B). Under these conditions, the *vtc4-1* mutant exhibited rates of chloroplast and mitochondrial electron transport that were comparable to those of WT cells (Fig. 4A). Furthermore, the TA-S–grown mutant was also able to induce ARS activity to a level similar to that of WT cells (Fig. 4, B and C). These results not only prove the role of VTC4 in controlling ATP homeostasis when the cells are deprived of S but also suggest that the elevated ATP in the mutant was responsible for the reduced rates of respiratory electron flow and PET and that the accumulation of polyP is not directly responsible for allowing cells to acclimate to S deprivation. In sum, the high level of intracellular ATP that accumulates in the *vtc4-1* mutant likely causes a slowing of respiratory electron flow and PET and suppression of the SdR.

### Electron flow in mitochondria or chloroplasts independent of ATP synthesis triggers expression of SdR genes

**Fig. 5:**
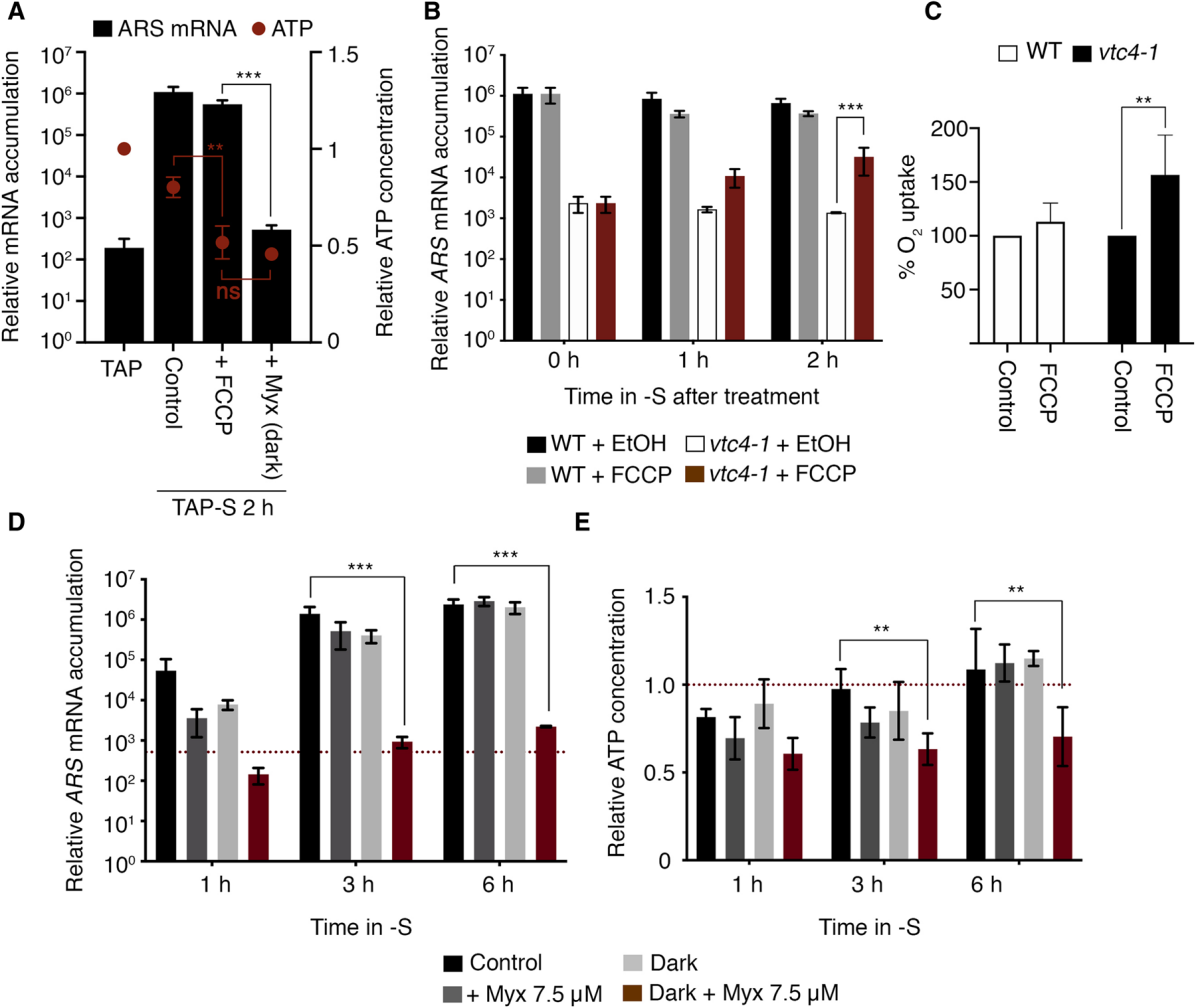
Reduction of electron transport in mitochondria and chloroplasts impairs acclimation to S deprivation in an ATP-independent manner. (**A**) Effect of FCCP and Myx + dark on ATP levels and *ARS* mRNA accumulation in the WT strain. Except when indicated, cultures were incubated in the presence of light. In all panels, “control” condition is S-depleted cells treated with ethanol, which is the solvent for FCCP and Myx. (**B**) Effect of FCCP on *ARS* expression in WT and *vtc4-1* after 6 hours in TAP-S. (**C**) Relative gross O_2_ uptake in WT and *vtc4-1* mutant treated with FCCP after 6 hours in TAP-S. FCCP values were normalized to the respiration rate in the presence of ethanol (control) for WT and the *vtc4-1* strains. (**D**) *ARS* mRNA accumulation and (**E**) ATP levels in S-deprived cells in the light (control, respiration, and photosynthesis active), in the dark (only respiration), in the light plus Myx (only photosynthesis), or in the dark plus Myx (respiration and photosynthesis inhibited). Dotted line shows the mRNA levels and ATP concentration in nutrient-replete medium. ATP value of 1 corresponds to 81.91 ± 8.05 pmol ATP μg chlorophyll^−1^. Error bars represent ±SD, *n* ≥ 3. Student’s *t* test was performed. **P values <0.01 and ***P < 0.001. “ns” means nonstatistically significant difference.

Inhibition of SdR in the *vtc4-1* mutant might be a consequence of diminished mitochondrial or chloroplast electron flow. During mixotrophic growth (TAP medium in the light), *Chlamydomonas* generates energy through both respiration and photosynthesis. To study the impact of electron flow in the organelles on the SdR, we deprived WT cells of S in the dark and added the respiration inhibitor Myxothiazol (Myx). Myx is a mitochondrial complex III inhibitor that blocks most mitochondrial electron flow and ATP synthesis. In the absence of mitochondrial and chloroplastic electron flow (Myx in the dark), there was no *ARS* induction, while under the same conditions without the addition of Myx, the cells exhibited normal acclimation (Fig. 5, A and D). These results suggest that eliminating electron flow in both mitochondria and chloroplasts prevents SdR induction. However, the ATP levels in dark maintained cells treated with Myx dropped by more than 50% (Fig. 5A), which could be the cause of the repressed SdR. To distinguish between the effect of reduced electron flow and the lower concentration of ATP on *ARS* expression, we used the uncoupler carbonyl cyanide *p*-trifluoromethoxyphenylhydrazone (FCCP). This protonophore, which has been previously used on *Chlamydomonas* (*37*, *38*), dissipates the proton gradient across the membranes, which prevents ATP synthesis in both mitochondria and chloroplasts without reducing the rate of electron transport. Myx in the dark and FCCP in the light both similarly lowered the ATP levels in WT cells (to ~50%; Fig. 5A). These two treatments had an opposite effect on *ARS* mRNA accumulation. In the presence of FCCP, which promotes electron transport, *ARS* was strongly induced, similar to WT control conditions. However, under the condition in which all electron transport was strongly reduced (Myx in the dark), *ARS* expression was almost completely repressed (Fig. 5A). These results indicate that the elevated level of ATP observed in the S-deprived *vtc4-1* mutant was not directly responsible for suppression of SdR, but SdR suppression was the consequence of the impact of high levels of ATP on electron transport. Under high (*vtc4-1* mutant in TAP-S) and low (*vtc4-1* mutant in TA-S and FCCP/Myx treatments) ATP conditions, the transcriptional activation of the SdR depended exclusively on active electron flow.

To further test whether the inability of the *vtc4-1* mutant to sustain SdR was the consequence of reduced electron transport, we treated WT and the *vtc4-1* mutant that had already experienced 6 hours of S deprivation with FCCP. After 6 hours in TAP-S, both respiration and photosynthesis were partially blocked in the mutant, and the level of *ARS* mRNA was declining (Figs. 1D and 4C). For WT cells, FCCP treatment barely affected *ARS* mRNA accumulation. However, FCCP-treated *vtc4-1* cells exhibited a notable increase (>20-fold) in *ARS* expression (Fig. 5B), which paralleled an elevated rate of respiration, indicated by a 50% increase in O_2_ uptake (Fig. 5C). Together, these data confirmed that the transcription of the SdR genes requires respiratory electron flow or PET independent of the ATP concentration, although other signals elicited by nutrient deprivation and probably sensed by plasma membrane receptors would also be required (*28*, *39*). Therefore, polyP synthesis has a vital role in maintaining active electron flow, which is necessary for the cells to acclimate to changing nutrient levels.

Last, we tested whether electron flow in both chloroplasts and mitochondria is needed to induce acclimation. We performed experiments in which we blocked electron transport in WT cells in only one organelle (Myx in the light to eliminate respiratory electron flow or only dark to eliminate PET). Under both of these conditions, we only observed a slight delay in *ARS* mRNA accumulation (Fig. 5D). Hence, electron flow in either organelle can generate the signal that induces and sustains *ARS* expression. Similar results were obtained when we used a complex III–deficient mutant instead of Myx (fig. S8), which suggests that the Myx effect is specifically due to its impact on mitochondrial electron transport. The delay in *ARS* induction observed when electron flow in only one of the organelles was blocked and the almost total loss of induction when both photosynthetic and respiratory electron flow was inhibited were also observed for *SULTR2*, *SLT1*, and *ECP76* (fig. S9), demonstrating that the SdR requires the integration of signals coming from either PET or respiratory electron flow.

### PolyP synthesis also affects cellular energetics and acclimation during nitrogen (N) deprivation

One of the highly induced genes in *Chlamydomonas* cells experiencing N deprivation is *LAO1*, which encodes l-amino oxidase (*40*). LAO1 is a periplasmic protein, similar to ARS, that scavenges amine groups from extracellular organic molecules (*41*). Aksoy *et al.* (*16*) previously reported that a *Chlamydomonas* *vtc1* mutant showed reduced induction of the LAO1 protein when cells were deprived of N. To study whether polyP synthesis is also required to maintain ATP homeostasis and sustain *LAO1* gene expression, we transferred the *vtc4* mutation to a genetic background able to assimilate all inorganic N (the original parental strain in this study, CMJ030, uses ammonium, but not nitrate, as a sole N source) by crossing the *vtc4-1* mutant and the WT strain 21gr (CC-1690), which can grow with either nitrate or ammonium as a sole N source; this is a more appropriate genetic background when examining N deprivation. We verified that the resulting mutant (vtc4-1*) in the new parental background showed the *ars^−^* phenotype when deprived of S and that we could rescue this phenotype by the introduction of a WT copy of the *VTC4* gene (fig. S10).

**Fig. 6:**
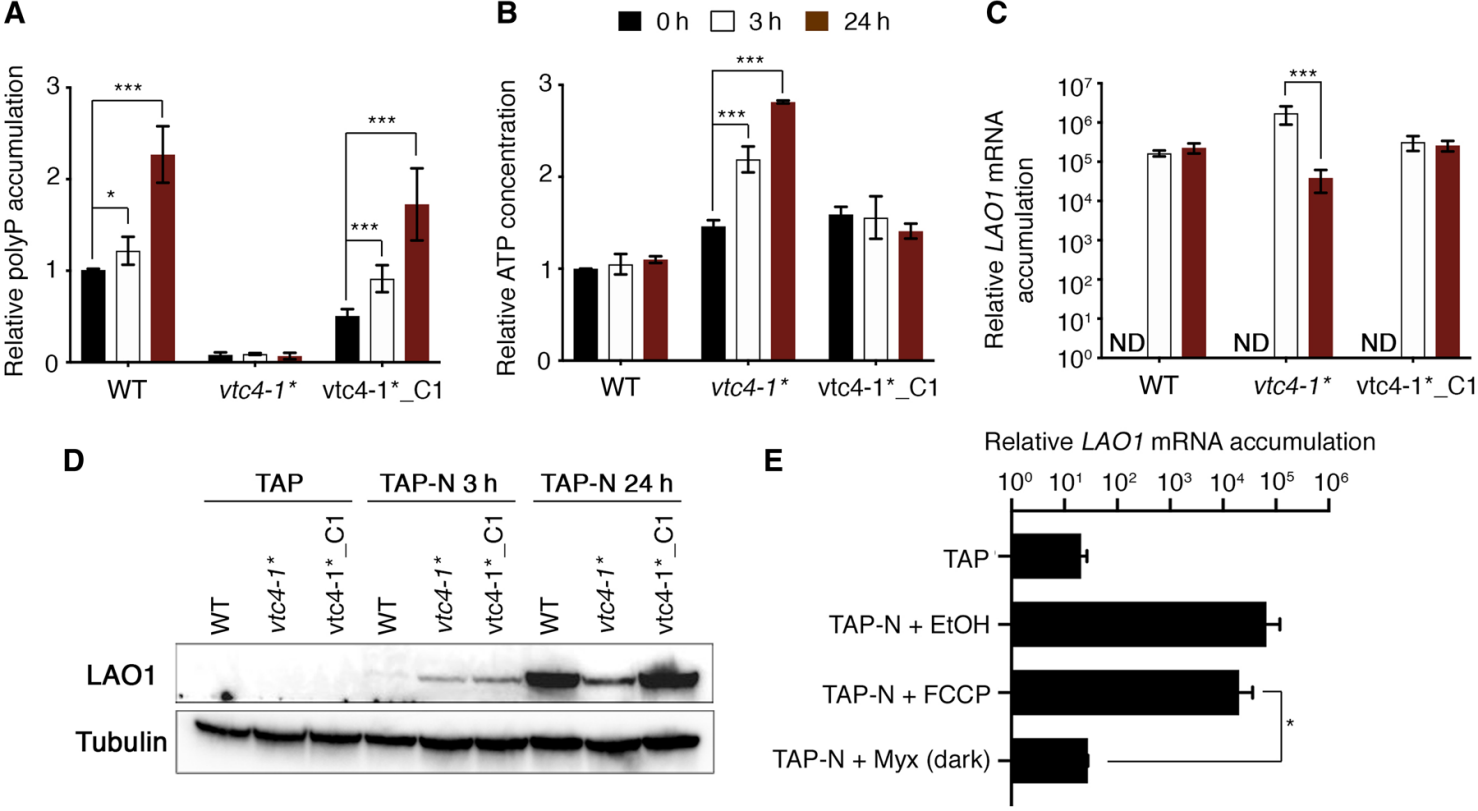
Links among polyP synthesis, electron flow, and acclimation are also conserved in N deprivation. (**A**) PolyP accumulation at various time following N deprivation, (**B**) ATP concentration, (**C**) *LAO1* mRNA levels, and (**D**) LAO1 protein levels in the WT (21gr), vtc4-1* mutant, and complemented strain (vtc4-1*_C1). The asterisk (*) indicates that the *vtc4-1* lesion was transferred into a WT genetic background for N assimilation (21gr; fig. S10). ND means “not detected,” and error bars represent ±SD, *n* ≥ 3. (**E**) Effect of FCCP and Myx + dark on *LAO1* mRNA accumulation in the WT strain. Except when indicated, cultures were incubated in the presence of light. PolyP and ATP values represented as 1 for 21gr corresponds to 313.33 pmol polyP μg chlorophyll^−1^ and 75.83 ± 28.18 pmol ATP μg chlorophyll^−1^, respectively.

As previously shown for S deprivation, polyP accumulated in the WT (21gr genetic background) and the complemented line, but not in the mutant lacking VTC4 (Fig. 6A). As expected, this inability to synthesize polyP impaired the capacity of the cells to buffer ATP levels, with the consequent increase in the intracellular ATP concentration (>2.5-fold higher) in vtc4-1* (Fig. 6B). This lack of polyP synthesis also led to substantial repression in LAO1 mRNA (~40-fold) and protein accumulation after 24 hours of N deprivation (Fig. 6, C and D). Last, similar to the results for S deprivation, treatment with FCCP and Myx in the dark showed that *LAO1* expression appears to be linked to electron transport independently of the ATP concentration (Fig. 6E). These results demonstrate a role for polyP synthesis in maintaining ATP homeostasis and sustaining the acclimation of cells to N deprivation.

## DISCUSSION

**Fig. 7:**
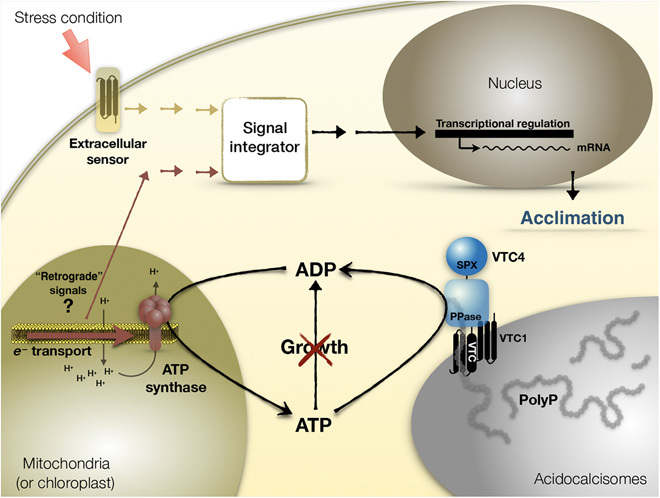
Model depicting integration of VTC complex and polyP synthesis in energetic regulation of cellular acclimation under stress conditions. Under S deprivation, when growth is blocked, acidocalcisomes accumulate energy in the form of polyP. The formation of polyP allows for both the regeneration of ADP and the maintenance of the ATP homeostasis, which sustain ATP synthesis and mitochondrial/photosynthetic electron flow. Electron flow in the energy-generating organelles is required to generate retrograde signals that are needed for the induction of acclimation responses (in this case, S or N deprivation). Similar regulation is likely to occur under other stress conditions. SPX, SYG1/Pho81/XPR1 domain; PPase, polyP polymerase.

Acclimation responses are energy consuming, and before being initiated, the cells must integrate not only external cues, including nutrient availability through plasma membrane sensors (*34*, *39*), but also they must respond to other signals that report the cell’s energetic status and potential for rapid acclimation. Thus, the cells would adjust their acclimation responses to energy availability. In this work, we demonstrate that the responses of *Chlamydomonas* to S and N deprivation are linked to polyP synthesis, ATP levels, and mitochondrial and chloroplast electron flow (Fig. 7). These connections provide a mechanistic/holistic view of the pleiotropic nature of the varied phenotypes associated with the inability of cells to synthesize polyP. Our studies indicate that the key to the SdR is not the polyP molecule itself but rather the synthesis of polyP in regulating ATP levels and maintaining electron transport activities that are critical for acclimation. Additional studies will be required to elucidate the impacts of these links on the cell’s capacity to cope with other stressful situations and to extrapolate the results presented here to other organisms. For example, our data raise the possibility that polyP-dependent virulence and survival of microorganisms (*5*, *13*, *42*) could be a consequence of the stabilization of ATP levels, which would sustain respiratory electron flow in the microbe during the first stages of infection. PolyP synthesis may also be essential to maintain active electron flow in the host as it acclimates to the stress associated with invasion. The virulence of trypanosomes, which affects millions of people in the world, is substantially affected by disruption of polyP synthesis (*42*). The VTC complex, the enzymatic machine in these protozoa that uses ATP to synthesize polyP, has been considered a potential target for the treatment of trypanosoma-caused diseases. Therefore, a detailed understanding of the links between polyP synthesis and cellular metabolism could allow for the development of new treatments to fend off parasites (*23*).

A major finding of our work is that polyP synthesis is not essential to induce acclimation of *Chlamydomonas* to S deprivation but that it is vital for sustaining this acclimation. The *vtc4-1* mutant, which is unable to synthesize polyP, exhibited acclimation only during the first 3 hours following the imposition of S deprivation, a period in which polyP was barely synthesized by WT cells. The role of polyP synthesis in sustaining the SdR appears to be the consequence of the capacity of the VTC complex to use ATP (storing the energy in the molecule as polyP), preventing high levels of its accumulation. The characterization of the *vtc4-1* mutant, which is unable to synthesize polyP, has allowed us to identify links among polyP accumulation, the regulation of intracellular ATP levels, and respiratory electron flow and PET. During S deprivation, cells are unable to grow, and the cellular energy demand drops markedly. However, as long as Pi and ADP are abundant, cells continue to synthesize excess ATP that is used to accumulate polyP. PolyP accumulation not only serves in the storage of energy and Pi but also enables the regeneration of ADP, which allows for continued respiratory electron flow and PET. Here, we clearly demonstrate a role of these electron transport activities in triggering and sustaining SdR; these findings suggest that the cells use electron flow as a proxy for energy availability. The inability to recycle ATP would lead to elevated ATP levels, which, in turn, retards both respiratory electron flow and PET and signals a compromised metabolic status. The growth of *Chlamydomonas* cells declines upon nutrient deprivation, leading to a reduced demand for energy, and yet, during nutrient deprivation, the transcripts and proteins associated with mitochondrial complexes are stable during mixotrophic growth (*28*, *40*), while the transcripts and proteins associated with photosynthetic complexes are stable during photoautotrophic growth (*43*–*45*). These observations are in accord with our conclusion that effective acclimation to S deprivation (and likely other deprivation responses) relies on active electron transport in the energy-related organelles. Moreover, polyP also appears to play a similar role in N deprivation; its synthesis also is required for normal acclimation to this deficiency. Together, our data indicate that the synthesis of the polyP polymer is crucial for the cells to cope with the energetic dynamics that occur under stress conditions.

A link between polyP and mitochondrial function has been previously shown in mammalian cells. Heterologous overexpression of a yeast exopolyphosphatase in mammalian cell lines, which degrades polyP, inhibits normal mitochondrial function, alters mitochondrial Ca^2+^ metabolism, and may affect the development of the mitochondria permeability transition pore (*46*). On the basis of our data, the polyP molecule itself is not directly required for mitochondrial function in S-deprived *Chlamydomonas* cells. The *vtc4-1* strain exhibited near full recovery of mitochondrial and chloroplastic electron flow when S and P were simultaneously removed from the medium (Fig. 4A), conditions that prevent accumulation of high ATP levels in the absence of polyP. On the basis of these physiological responses and our pharmacological results, we conclude that it is the synthesis of polyP that is critical for maintaining mitochondrial and chloroplastic electron transport by controlling the cellular ATP concentration.

While electron transport is one key regulatory component of acclimation, the integration of this component with signals that directly control SdR-associated genes requires further investigation. However, previous research has established the involvement of SNRK protein kinases (SNF1-related protein kinase) in the regulation of the SdR (*47*). These kinases in plants serve to integrate metabolic signals elicited by abiotic stress with acclimation responses (*48*). *Chlamydomonas* SNRK2.1 is essential for inducing transcription of most genes required for acclimation of the cells to S deprivation (*28*). Whether signals generated by electron flow in chloroplasts and mitochondria (e.g., reactive oxygen species or nitric oxide) modulate the activity of this kinase is not known. In plants, both phosphorylation and nitrosylation can alter the activity of some SNRK proteins (*49*). We raise the possibility that the SNRK proteins associated with S deprivation in *Chlamydomonas* (*47*) integrate signals coming from the environment (e.g., through phosphorylation) with intracellular indicators of energetic and metabolic conditions (e.g., through nitrosylation). Mitochondria and chloroplasts are hubs for generating retrograde stress signals that affect gene expression through redox signaling and the production of reactive oxygen species. Recently, it was reported that *Chlamydomonas* cells accumulate NO in response to S deprivation (*43*), but in-depth studies are required to determine whether or not this signaling molecule participates in the acclimation process.

As established in this study, polyP synthesis is required to maintain SdR after 2 to 3 hours in starvation, and this synthesis is likely activated by the binding of inositol pyrophosphate to the SPX domain of VTC4. We speculate that once the cells experience nutrient limitation (before polyP synthesis), inositol phosphate metabolism could serve as the first “buffer” to control ATP levels. As cellular ATP increases, the most highly phosphorylated forms of inositol, the inositol pyrophosphates Ins7P and Ins8P, are synthesized. These two molecules bind to the VTC4 SPX domain, activating polyP synthesis (*10*), although with different efficiencies. Ins8P has been shown to be the most potent VTC4 activator in yeast (*11*). Therefore, we hypothesize that an initial increase in intracellular ATP would elicit Ins7P accumulation and partial activation of VTC4. With a further increase in the ATP concentration, Ins8P begins to accumulate, and VTC4 becomes fully active, providing cells a higher capacity to buffer ATP. The connection between inositol metabolism and polyP synthesis has been reported in trypanosomes and yeast (*10*, *50*), and a link between inositol pyrophosphates and the control of ATP homeostasis has also been reported in yeast. The *kcs1* yeast mutant, which is unable to synthesize inositol pyrophosphate, accumulates ATP and exhibits a reduction in mitochondrial activity (*51*). Our data raise the possibility that this increase in ATP could reflect the inability of this mutant to activate the SPX domain of VTC4 and the initiation of polyP synthesis. This mechanism of regulation would allow for a tiered ATP buffering capacity based on the sequential activation of inositol pyrophosphate and a subsequent elevation in polyP synthesis. Starch and lipids synthesis may also function in ATP buffering and maintenance of mitochondrial and chloroplast electron flow when the capacity to store polyP in acidocalcisomes is exceeded.

How an increase in the ATP concentration in S-deprived *vtc4-1* mutant cells inhibits electron flow is not absolutely clear, although part of the response could reflect limited availability of substrate (ADP and Pi) for sustaining high levels of electron transport in both mitochondria and chloroplasts. In addition, in higher eukaryotes, high ATP levels allosterically regulate complex IV in mitochondria (*35*), while in bacteria, the ATP synthase is regulated by the ATP/ADP ratio (*36*). Our studies demonstrate that *vtc4-1* mutant cells were affected in their total ATP concentration, the ATP/ADP ratio, and the levels of respiratory electron flow and PET. Furthermore, the ATP level and ATP/ADP ratio in the *vtc4*-*1* strain exhibited a similar increase, with no change in the ADP concentration. This result suggests that *Chlamydomonas* cells could either induce purine synthesis (elevated intracellular adenosine phosphate pool) or promote the phosphorylation of AMP to compensate for reduced regeneration of ADP through the polyP kinase. Over the short term, this strategy would allow cells to maintain basal electron flow rates (50% of respiratory electron flow and PET), which could be essential for basic organelle functions and survival.

Overall, the aspects of polyP regulation described in this manuscript could extend well beyond S deprivation and nutrient deprivation responses and into modulation of cellular processes in response to fluctuations in environmental conditions that alter growth and elicit changes in the levels of the different adenylate species. PolyP, a polymer that had been experimentally neglected, appears to be an integral factor in controlling a cascade of energetic processes that enable dynamic acclimation of organisms to fluctuations of the natural environment.

## MATERIALS AND METHODS

### *Chlamydomonas* and yeast strains

CMJ030 (CC-4533) was used as our WT strain and is the parental strain of the *vtc4-1* (LMJ.RY0402.086990) and *vtc4-2* (LMJ.RY0402.195991) mutants, which were generated by insertional mutagenesis and are strains in the CLiP (Chlamydomonas Library Project) mutant library (*32*). The *dum11* mutant (CC-4098, affected in complex III of the respiratory chain) was obtained from the Chlamydomonas Resource Center (www.chlamycollection.org/), and 137c (CC-124) was used as the parental strain. 21gr (CC-1690) was the WT strain for the experiments in which cells were exposed to N deprivation.

### Media and culture conditions

TAP medium was prepared according to standard recipes available at www.chlamycollection.org/methods. S-depleted TAP (TAP-S) medium was prepared by substitution of the sulfate salts with chloride salts. For P- and S-free (TA-S) medium, potassium phosphate was replaced with potassium chloride (*52*). Ammonium-free TAP (TAP-N) medium was used for N deprivation experiments. Hutner’s trace elements for TAP and TAP-S media were purchased from the Chlamydomonas Resource Center. The solid medium contained agar (15 g liter^−1^). *Chlamydomonas* cells were grown in Erlenmeyer flasks at 23°C with agitation under fluorescent tubes at 100 μmol photons m^−2^ s^−1^. For nutrient deprivation experiments, cells were grown in TAP medium, pelleted by centrifugation for 2 min (2300*g*), and washed once in TAP-S medium before resuspending the cells at 10 μg ml^−1^ of chlorophyll in the appropriate medium.

### Reagents

Myx and FCCP from Sigma-Aldrich were dissolved in 100% ethanol and used at 7.5 and 5 μM, respectively.

### ARS activity and chlorophyll determination

Cells growing on solid TAP medium were streaked and maintained on TAP-S agar for 5 days. ARS activity was assayed by spraying colonies with 10 mM 5-bromo-4-chloro-3-indolyl sulfate (Sigma-Aldrich) in 0.1 M tris-HCl (pH 7.5) as previously described (*47*); the blue signal appeared after 1 to 2 hours. In liquid medium, ARS activity was assayed by mixing 500 μl of culture (10 μg ml^−1^ chlorophyll) with 500 μl of the reaction mixture [4.5 mM *p*-nitrophenyl sulfate as substrate, 10 mM imidazole, and 0.1 M glycine (pH 9.0)]. Incubation of the reaction was for 30 min at 30°C. The reaction was stopped with 0.2 N NaOH, the debris pelleted by centrifugation for 2 min (14,000*g*), and the absorbance of the supernatant at 405 nm was determined. Chlorophyll measurements were made as previously described (*53*) after extraction of the pigments with methanol.

### RNA extraction and quantitative reverse transcription polymerase chain reaction

Total RNA was isolated using phenol/chloroform (pH 4.5) and LiCl. Residual DNA was removed by TURBO DNase (Thermo Fisher Scientific). A detailed protocol for RNA extraction and deoxyribonuclease treatment were previously described (*54*). First-strand complementary DNA (cDNA) was generated by reverse transcription of 0.5- to 1.0-μg total RNA using the iScriptTM Reverse Transcription Supermix (Bio-Rad). Real-time polymerase chain reaction (PCR) was performed in the Roche LightCycler 480 with the SensiFastTM SYBR No-Rox Kit (Bioline), as described by the manufacturer. The samples generated by first-strand cDNA synthesis were diluted 2.5×, and 1 μl was used as a template for the PCRs. Two-step cycling conditions were used: 2 min at 95°C, followed by 40 cycles of 95°C for 5 s, 60°C for 30 s, and quantification of the fluorescence at the end of each cycle. Melting curve analyses (60° to 100°C, with a heating rate of 0.5°C s^−1^ and continuous fluorescence measurements) and electrophoretic analyses on agarose gels were performed to determine the specificity of the amplification products. The *CBLP* gene was used as the housekeeping control, and relative fold differences were calculated on the basis of the ΔCt method [2 − (Ct target gene − Ct CBPL)]. The primers used for quantification were RT-ARS2fw (GCTCAGGACGAAACCATCAAG) and RT-ARS2rev (ATCGGTGAAGATCACCACAAAG) for ARS (Cre16.g671350); RT-SULTR2fw (ACGTGGCATGCAGCTCAT) and RT-SULTR2rev (CTTGCCACTTTGCCAGGT) for SULTR2 (Cre17.g723350); RT-SLT1fw (ACGGGTTCTTCGAGCGAATTGC) and RT-SLT1rev (CGACTGCTTACGCAACAATCTTGG) for SLT1 (Cre12.g502600); RT-ECP76fw (CCTCGCTCTCCTCGCTGCTG) and RT-ECP76rev (CGGCCGACTTGGGTAATTGC) for ECP76 (Cre06.g288550); RT-ACT1fw (CTCTGGTGTGCGACAATGGTTC) and RT-ACT1rev (TCGCCAACGTACGAGTCCTTC) for ACT1 (Cre13.g603700); RT-GPX5fw (ACCAATCGCCTAACACCTGTGC) and RT-GPX51rev (TTGTGGCGTAAAGCCGCACTTG) for GPX5 (Cre10.g458450); RT-LAO1fw (GAGACTGTGATGCCCAAAAAGTG) and RT-LAO1rev (GCTTGCCCAGGCCGCGAATGGAA) for *LAO1* (Cre12.g551352); and RT-CBLPfw (CAAGATCTGGGACCTGGAGAGC) and RT-CBLPrev (CTGGGCATTTACAGGGAGTGG) for CBLP (Cre06.g278222).

### Genotyping of *vtc4*

Characterization of the insertion sites in *vtc4-1* and *vtc4-2* mutants was performed as detailed on the website www.chlamylibrary.org/help. DNA was extracted from fresh colonies grown in TAP medium and resuspended and vortexed in 50 μl of 5% Chelex-100 (Bio-Rad). The resuspended cells were boiled for 8 to 10 min, cooled on ice for 1 min, and made homogeneous by vortexing the suspension for 10 s. Cells were then pelleted by centrifugation for 1 min (20,000*g*), and 2 μl of the supernatant were used as the template for PCR, which was performed using Taq Polymerase from QIAGEN according to the following protocol: 95°C for 3 min, 40 cycles of 95°C for 20 s, 60°C for 20 s, and 72°C for 2 min. Primers used, indicated in figs. S2 and S3, were oMJ913 (GCACCAATCATGTCAAGCCT), oMJ944 (GACGTTACAGCACACCCTTG), 1 (CCGCTCTCCAGATCCCCTTTGAC), 2 (GCTAGAGCCTGGGCGGGTAGA), 3 (GAAGGGAAACGGAGGAAATGAGC), and 4 (TACGCCATCCTCGTCAGCACAG).

### Complementation of *vtc4-1* mutant

The bleomycin cassette from the pSP124S plasmid was replaced by a hygromycin resistance cassette (from pRAM118 vector) using the restriction sites Kpn I–Hind III and generating the vector pSP124SH. The full cDNA (2337 bp) for *VTC4* was amplified with primers VTC4-NdeIfw (GACATATGAAGTACGGAAAGTATATTGAG) and VTC4-EcoRIrev2 (CGAATTCTAGAACAGCGACAGCGTCCC) using 0.4 U of Phusion Hot Start II DNA Polymerase with 0.42 M dimethyl sulfoxide and 0.6 M betaine. PCR conditions were those recommended by the manufacturer, and the annealing temperature was 63°C. The cDNA was cloned into the Nde I–Eco RI sites of pBleJM43. To introduce the native promoter and the first intron of *VTC4*, the promoter (920 bp), 5′ untranslated region (5′UTR), first two exons and 91 bp of the second intron were amplified with the primers VTC4-PHindIIIIfw (AAGCTTGCCGGCGGGACCACAGCTACGAGG) and VTC4g-653rev (CCGCCTACTTGCCAAGCACCTACG) using the same conditions as described above. This amplified fragment was cloned into Hind III–Afl II sites in the vector containing the full-length cDNA. Last, this construct was digested with Hind III and Not I, and the fragment generated cloned into pSP124SH. The final construct pSP124SH+P+*VTC4* bore the native promoter, 5′UTR, the cDNA of *VTC4* with the first intron, and the 3′UTR of the *PSAD* gene from pBleJM43. The *vtc4-1* mutant was transformed by electroporation with 2 μg of pSP124SH+P+*VTC4* linearized with Not I. GeneArt MAX Efficiency Transformation Reagent for algae (Invitrogen) was used for electroporation following the recommendations of the manufacturer. Transformants were selected on solid TAP medium containing hygromycin (20 μg ml^−1^; Enzo Life) and then assayed for ARS activity.

### PolyP quantification

A volume of *Chlamydomonas* culture containing 50 μg of chlorophyll was collected by centrifugation for 1 min (3000*g*), and polyP was extracted by the neutral phenol/chloroform and the ethanol precipitation method previously described (*55*). The lysates containing polyP were diluted (1:1000 to 1:4000) and quantified using the MicroMolar Polyphosphate Assay Kit (ProFoldin, PPD1000). For negative staining with 4′,6-diamidino-2-phenylindole (DAPI) (*56*), polyP samples were mixed with loading buffer [6× tris boric acid-EDTA (TBE), 15% Ficoll, 0.025% xylene cyanol FF, and 0.025% bromophenol blue] and electrophoresed into a 15% Criterion TBE-urea precast gel (Bio-Rad). The gel was rinsed with a fixative solution (50 mM tris base, 25% methanol, and 5% glycerol) and incubated for 30 min in staining solution [fixative solution + PureBlu DAPI (2 μg/ml), Bio-Rad]. After the gel was incubated for 1 hour in fixative solution to eliminate excess DAPI, it was exposed to ultraviolet light for 5 to 10 min. DAPI bound to polyP bleaches in minutes, while the fluorescence of free DAPI or DAPI bound to nucleic acid is stable.

### Quantification of ATP and ATP/ADP ratio

*Chlamydomonas* cells containing 50 μg chlorophyll were pelleted from cultures and resuspended in 925 μl of 0.5 M tris-HCl buffer (pH 7.5). A total of 75 μl of 70% perchloric acid (Sigma-Aldrich) was added to the cell samples, which were then vortexed for 15 s to both lyse the cells and deproteinize the lysate. The samples were then incubated on ice for 1 min, neutralized with 4 M KOH, mixed thoroughly by vortexing, incubated for an additional minute on ice, and then centrifuged for 1 min (3200*g*). A total of 500 μl of supernatant was transferred to a prechilled tube that was then flash-frozen in liquid N_2_ and stored at −80°C. A total of 25 μl of the supernatant was used for ATP quantification based on the “ATP Fluorometric Assay Kit” from Sigma-Aldrich (MAK190). Fluorescence was quantified in a Tecan Safire plate reader. For measuring the ATP/ADP ratio, we diluted the same samples (1:250) and used the “ADP/ATP Ratio Assay kit” (Sigma-Aldrich, MAK135). Bioluminescence was determined using the TD-20/20 luminometer from Turner Designs.

### Respiration measurements

Dark respiration and gross O_2_ uptake were measured using a Clark-type electrode with the Chlorolab 2^+^ System (Hansatech). *Chlamydomonas* cells in midlog growth phase were pelleted for 2 min (2300*g*), dispersed in TAP or TAP-S at 10 μg ml^−1^ chlorophyll, and then grown for 6 hours in the light. The cells were then dark-acclimated for 10 min, and 2 ml of culture was used to measure O_2_ uptake. Respiration values were calculated from the initial slope of dark O_2_ uptake. When indicated, gross O_2_ uptake was measured directly from light-incubated cells without dark acclimation. Cells were illuminated in the O_2_ chamber with light-emitting diode (LED) lighting (white light of 4100 K provided with the Chlorolab 2^+^ System) of the intensities indicated in the text.

### Photosynthetic electron transport

Cells grown in TAP or TAP-S media to a chlorophyll concentration of 10 μg ml^−1^ were dark-acclimated for 20 min. Chlorophyll fluorescence measurements were performed using a DUAL-PAM-100 (Walz). Fv/Fm was measured following dark acclimation. Cells were then exposed to 120 μmol photons m^−2^ s^−1^ for 15 s to quantify the yield of PSII. PET was calculated according to the formula PET = 0.5 × PSII yield × 0.84 × photosynthetically active radiation (in μmol photons m^−2^ s^−1^).

### *Chlamydomonas* genetic crosses

For the N deprivation experiments, the *vtc4-1* mutant was crossed, according to a previously described method (*57*), with a WT strain able to use ammonium, nitrate, or nitrite as sole N sources (strain 21gr; fig. S10). Instead of tetrad analysis, the zygotes were germinated in paromomycin (25 μg ml^−1^) and 7.5 mM nitrate as the sole N source, providing a positive selection for segregants that are drug resistant (containing the introduced cassette) and that can grow on nitrate; these segregants contain the *vtc4-1* lesion in the 21gr genetic background with respect to N utilization.

### Protein extraction and immunoblot analysis

Total cells in a volume of culture containing 50 μg of chlorophyll were pelleted for 2 min by centrifugation (2300*g*). The pellet was resuspended in protein extraction buffer [5 mM Hepes (KOH; pH 7.5), 10 mM EDTA (NaOH; pH 7.5), 1 mM phenylmethylsulfonyl fluoride, 1 mM ε-amino-*n*-caproic acid, and 1 mM benzamidine HCl], collected again by centrifugation for 2 min (14,000*g*), and resuspended in extraction buffer containing 100 mM dithiothreitol, 100 mM Na_2_CO_3_, 2% (w/v) SDS, and 12% (w/v) sucrose. Samples were boiled for 1 min, briefly incubated on ice, and then subjected to SDS–polyacrylamide gel electrophoresis (12% acrylamide gels); the loading of the samples were normalized to chlorophyll content (and similar levels of tubulin). The polypeptides were then transferred to polyvinylidene difluoride membranes using the Trans-Blot Turbo Midi and the Trans-Blot Turbo Transfer Systems from Bio-Rad following the manufacturer’s instructions. Membranes were blocked for 1 hour at room temperature with a 5% (w/v) suspension of powdered milk in tris-buffered saline containing 0.1% (w/v) Tween 20 (TBS-T). The membranes were then incubated for 2 hours with the primary LAO1 antibody (1:10,000, 3% milk in TBS-T) at room temperature followed by three 5-min washes in TBS-T and incubated with the secondary antibody (3% milk in TBS-T) for 1 hour at room temperature. Secondary antibodies used for LAO1 and tubulin were horseradish peroxidase (HRP)–conjugated anti-rabbit immunoglobulin G (IgG; Life Technologies, 656120) and anti-mouse IgG (Life Technologies, G2104), respectively. The membranes were then each washed three times for 5 min with TBS-T, and peroxidase activity was assayed by chemiluminescence using the WesternBright ECL HRP substrate (Advansta).

## Supplementary Material

abb5351_SM.pdf

## SUPPLEMENTARY MATERIALS

Supplementary material for this article is available at http://advances.sciencemag.org/cgi/content/full/6/40/eabb5351/DC1

## Appendix

View/request a protocol for this paper from *Bio-protocol*.
