# Supplementary material for: Metabolic control of acclimation to nutrient deprivation dependent on polyphosphate synthesis
Source: Sci Adv. 2020 Sep 30;6(40):eabb5351. doi: 10.1126/sciadv.abb5351 (PMC7556998; doi:10.1126/sciadv.abb5351)
Supplement: abb5351_SM.pdf [file abb5351_SM.pdf]

Science Advances
AAAS

advances.sciencemag.org/cgi/content/full/6/40/eabb5351/DC1

Supplementary Materials for

Metabolic control of acclimation to nutrient deprivation dependent on polyphosphate synthesis

E. Sanz-Luque*, S. Saroussi, W. Huang, N. Akkawi, A. R. Grossman*

*Corresponding author. Email: esanzluque@carnegiescience.edu (E.S.-L.); agrossman@carnegiescience.edu (A.R.G.)

Published 30 September 2020, *Sci. Adv.* **6**, eabb5351 (2020)
DOI: 10.1126/sciadv.abb5351

This PDF file includes:

Figs. S1 to S10

Supplementary Materials
S1

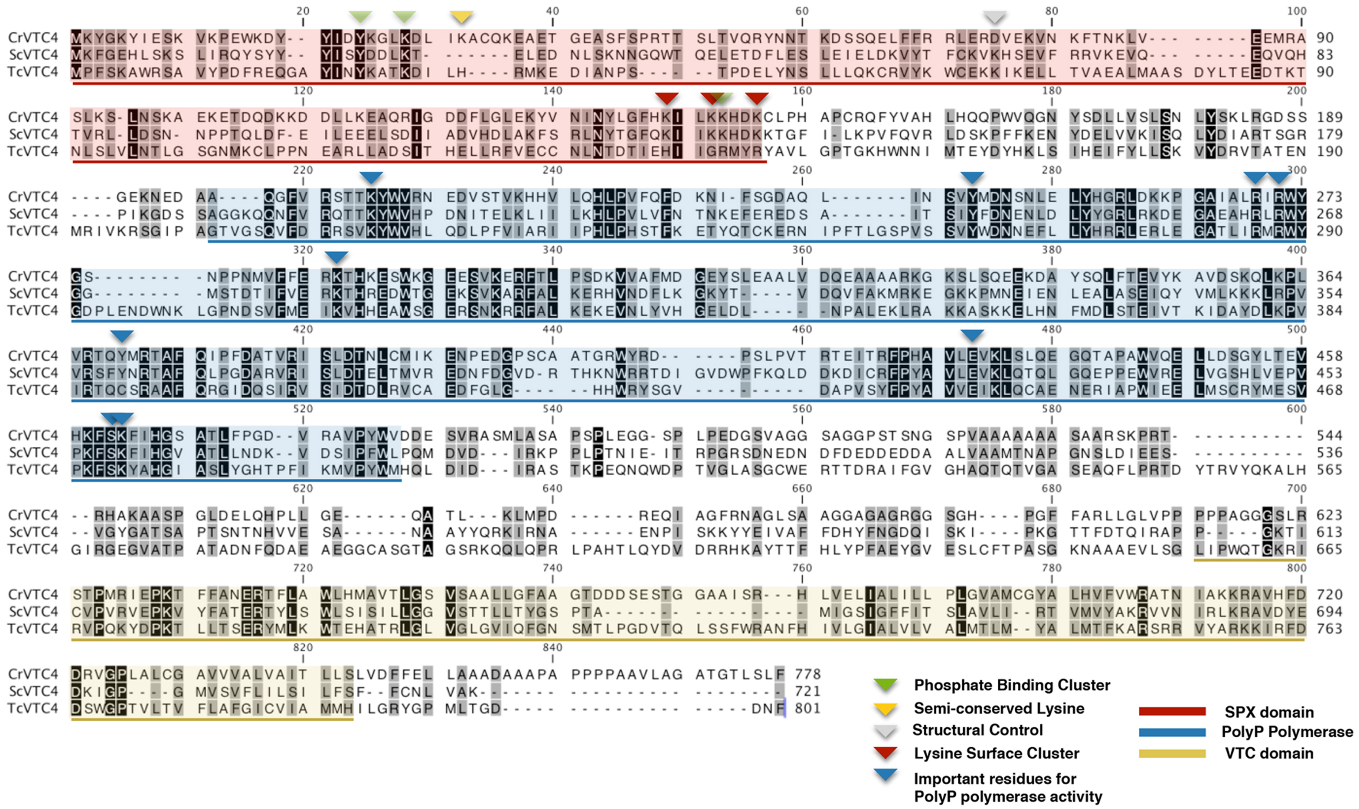

Supplementary Fig. 1. Alignment of the amino acid sequences of the VTC4 proteins. Protein sequences of *Chlamydomonas reinhardtii* (CrVTC4, PNW79162.1), *Sacharomycces cerevisiae* (ScVTC4, NP_012522.2) and *Trypanosoma cruzei* (TcVTC4, AHF82022.1) were aligned using the CLC Sequence Viewer. Coloured shaded boxes highlight the SPX (red), polyP Polymerase (blue) and VTC (yellow) domains. Coloured triangles indicate critical functional residues described in yeast (*7*, *9*).

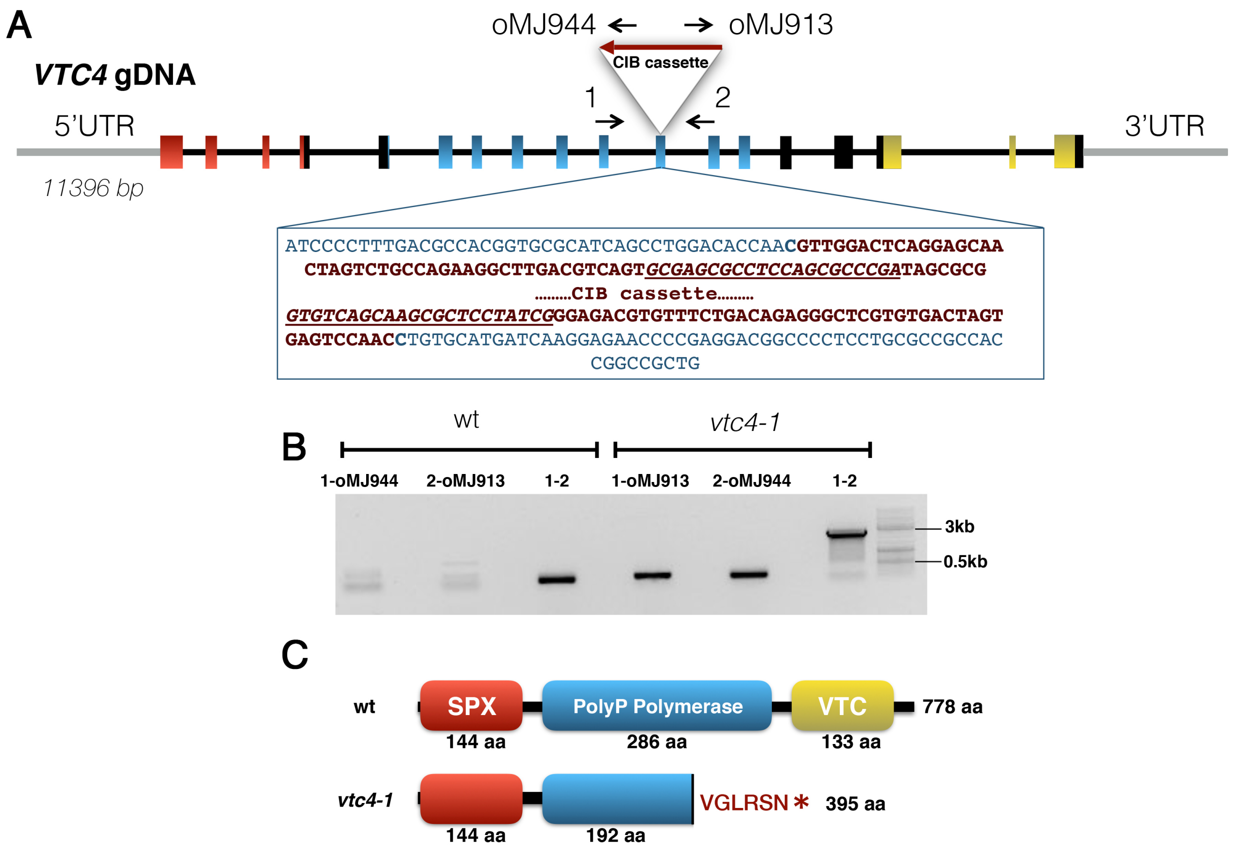

Supplementary Fig. 2. Schematic representation of *VTC4* gene and characterization of insertion in the *vtc4-1* mutant. (**A**), Model of *VTC4* gene. Grey and black lines represent UTRs and introns, respectively, whereas rectangles depict exons. Red, blue and yellow exons correspond to sequences encoding SPX, polyP polymerase and VTC domains, respectively. The long red arrow represents the inserted DNA (cassette) in *vtc4-1* and the short black arrows indicate the positions of the primers used for genotyping the mutant. The sequence provides a detailed view of the insertion site, with exon and cassette sequences in blue and red, respectively. Underlined, italicized bases in red correspond to the ID barcodes of the insertion in this strain, which are part of the inserted sequence separated by the CIB cassette (*22*). (**B**), Genotyping of wt (CMJ030) and *vtc4-1* strains. PCR bands were obtained with the indicated primers using gDNA as the template. (**C**), wt VTC4 protein and the truncated protein predicted to be synthesized in the *vtc4-1* mutant. The asterisk represents the first in frame stop codon.

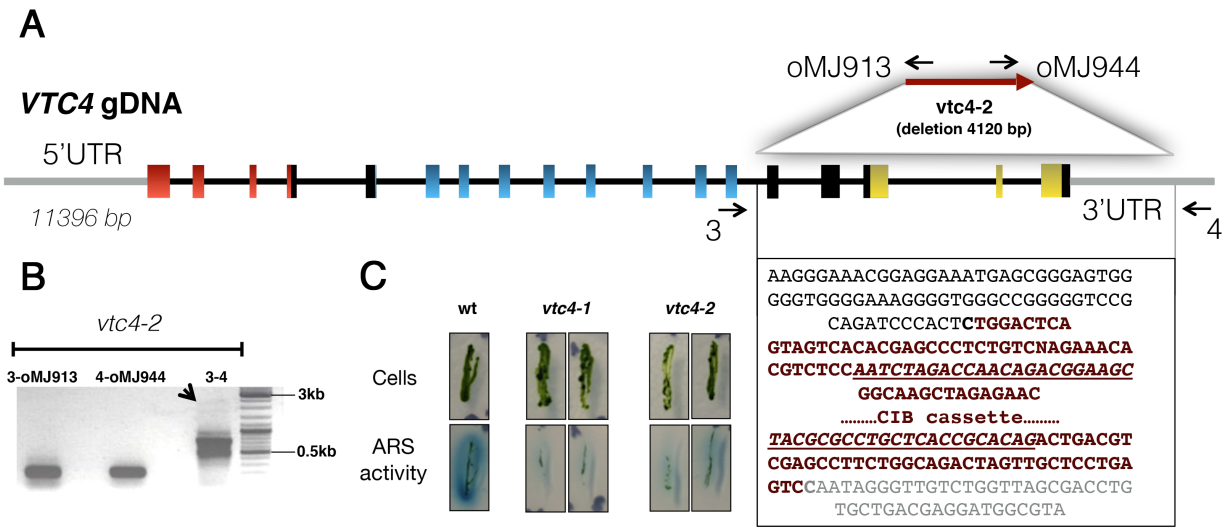

Supplementary Fig. 3. Characterization of insertion and ARS activity in *vtc4-2* mutant. (**A**), Model of *VTC4* gene. Grey and black lines represent UTRs and introns, respectively, whereas coloured rectangles depict exons, as described in **Supplementary Fig. 2**. The inserted DNA, primer positions and the sequence of the insertion site are also as in **Supplementary Fig. 2**. (**B**), Genotyping of *vtc4-2* strain. PCR bands were obtained with the indicated primers using gDNA as the template. (**C**), ARS activity of wt, *vtc4-1* and *vtc4-2* after 5 days of growth on TAP-S medium. Photo credits for (C): Emanuel Sanz-Luque, Carnegie Institution for Science.

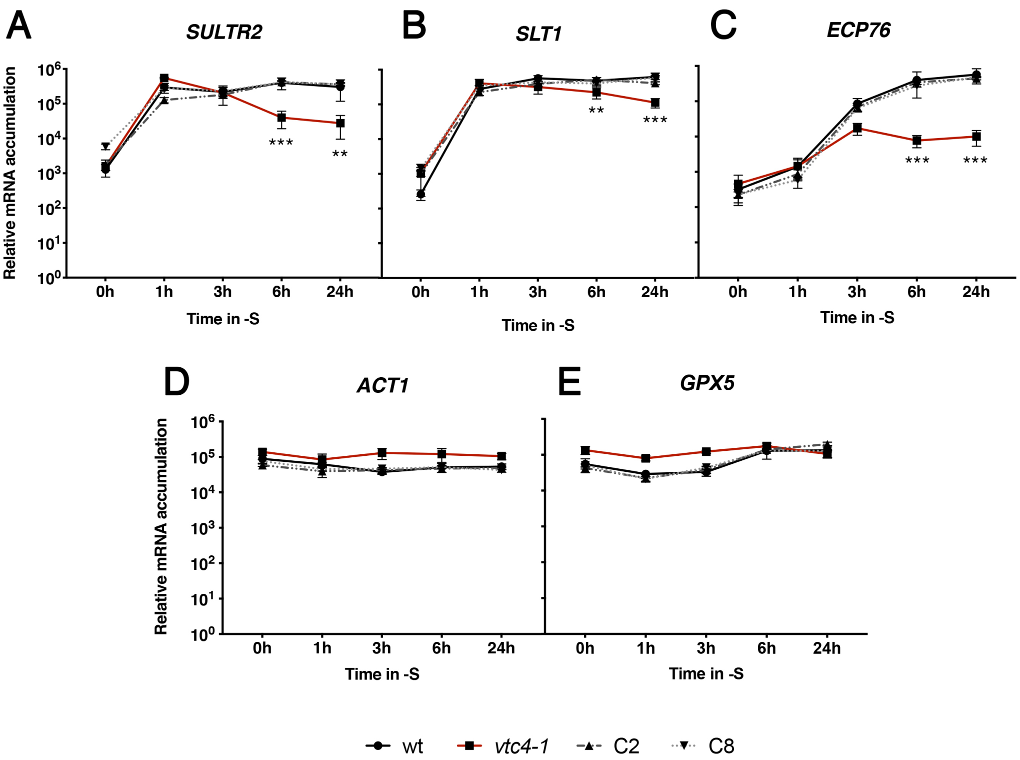

Supplementary Fig. 4. VTC4 activity impacts expression of other S deprivation inducible genes. Cells were grown in TAP medium, washed and transferred to TAP-S medium at a concentration of 10 µg ml^{-1} of chlorophyll. Relative accumulation of (**A**), *SULTR2*, (**B**), *SLT1*, (**C**), *ECP76*, (**D**), *ACT1* and (**E**), *GPX5* mRNAs was quantified at the indicated times following imposition of S deprivation by quantitative PCR using the *CBLP* transcript as the housekeeping control. Relative accumulation of *ACT1* (actin) and *GPX5* (glutathione peroxidase) mRNAs was quantified as controls to determine if the mutant is generally impacted for cellular transcript levels. Error bars represent ±SD n≥3. Student's t-test was performed, p values <0.01 (**) and <0.001 (***). "ns" means non statistically significant difference.

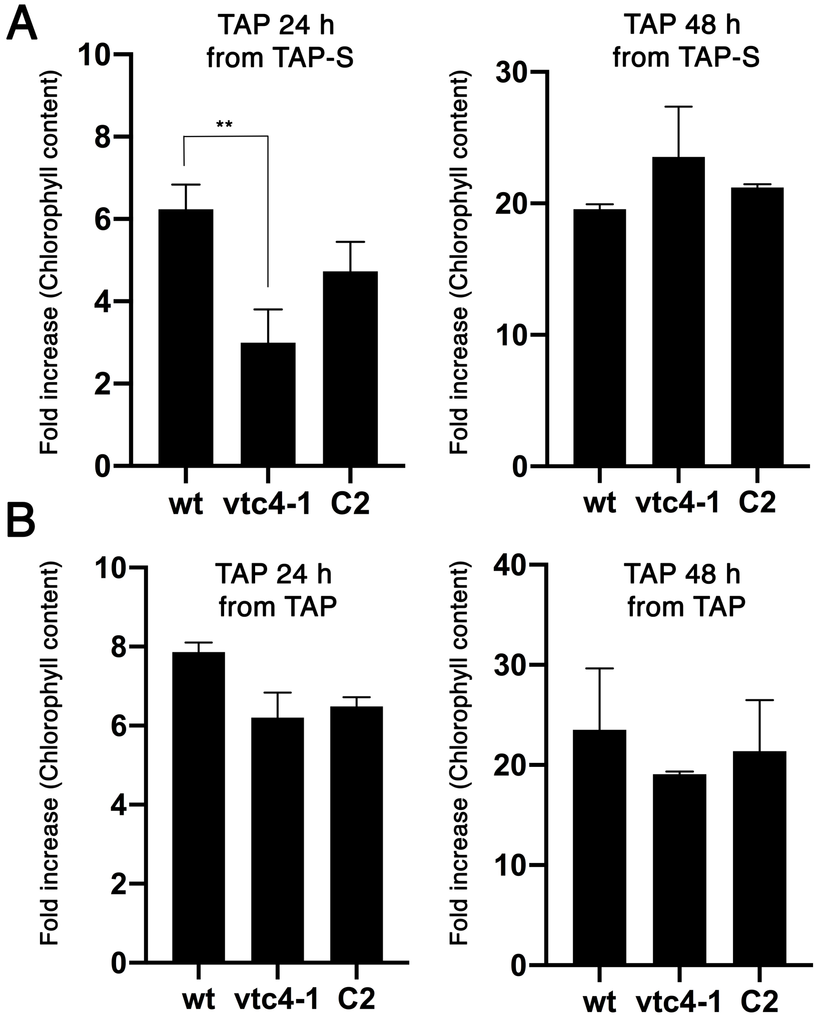

Supplementary Fig. 5. ***vtc4-1*** **growth after S replenishment. Cells depleted of** S for 24 h were inoculated to a final density of 2 µg/ml of chlorophyll in TAP medium and the increase in chlorophyll content was quantified. As a control, we transferred the cells that had been growing in TAP medium to the same nutrient replete medium.

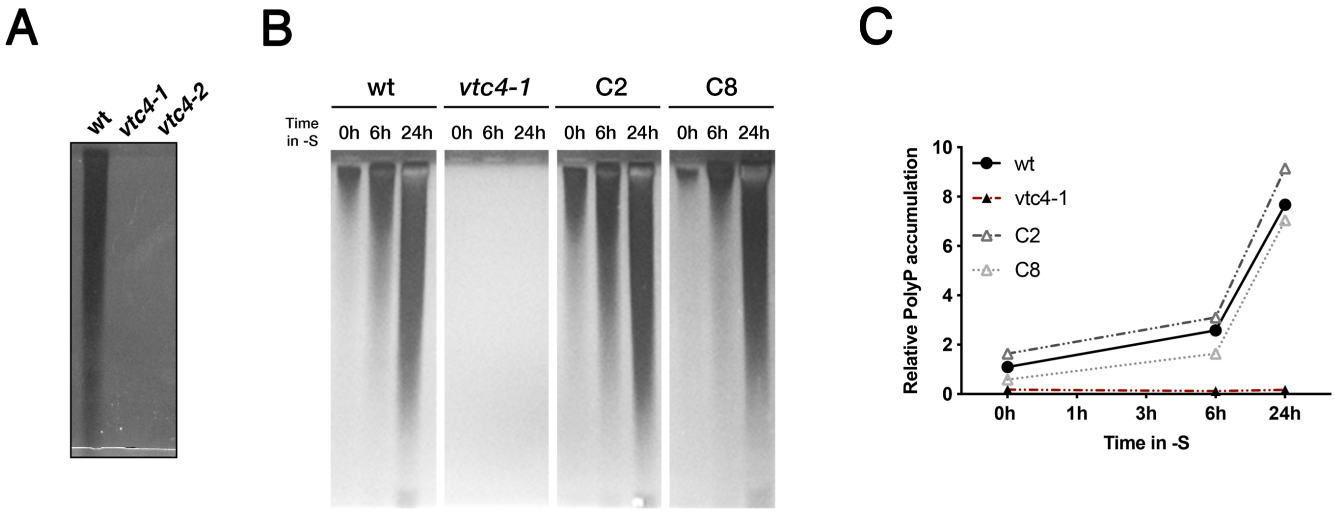

Supplementary Fig. 6. PolyP accumulation in nutrient replete conditions and during acclimation to S deprivation. (**A**), PolyP detection by PAGE analysis and negative staining of extracts from wt (CMJ030), *vtc4-1* and *vtc4-2* maintained in nutrient replete conditions. (**B**), PolyP detection by PAGE analysis and negative staining with DAPI in wt (CMJ030), *vtc4-1* and the complemented strains C2 and C8 at the indicated times after the imposition of S deprivaton. PolyP samples extracted from cells containing 50 µg chlorophyll were diluted 10 times before loading the samples onto the gel. (**C**), Relative polyP quantification of the same samples was performed using the “MicroMolar Polyphosphate Assay Kit” (ProFoldin).

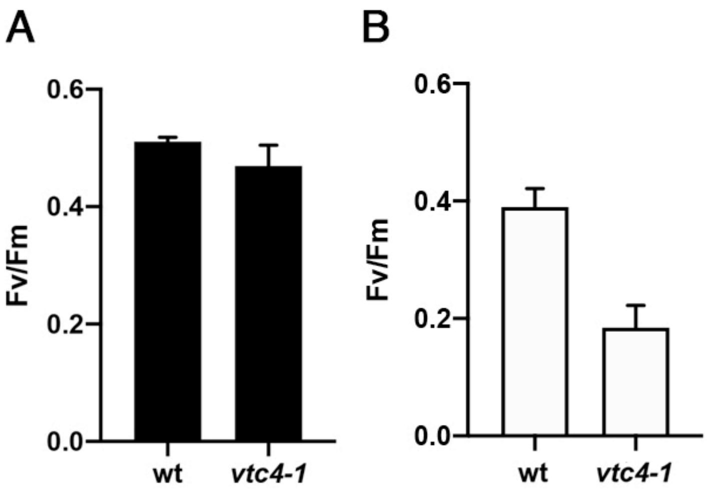

Supplementary Fig. 7. Photosystem II yield (Fv/Fm) after 6 h of S deprivation. wt and *vtc4-1* cells were exposed to S deprivation conditions for 6h. Before measuring the Fv/Fm, the cells were acclimated in the dark for 20 min. Fv/Fm values were measured before (**A**), and after (**B**), the illumination of the cells with actinic light (120 µmol photons · m^{-2} · s^{-1}) for 15 s. Error bars represent ±SD n≥3

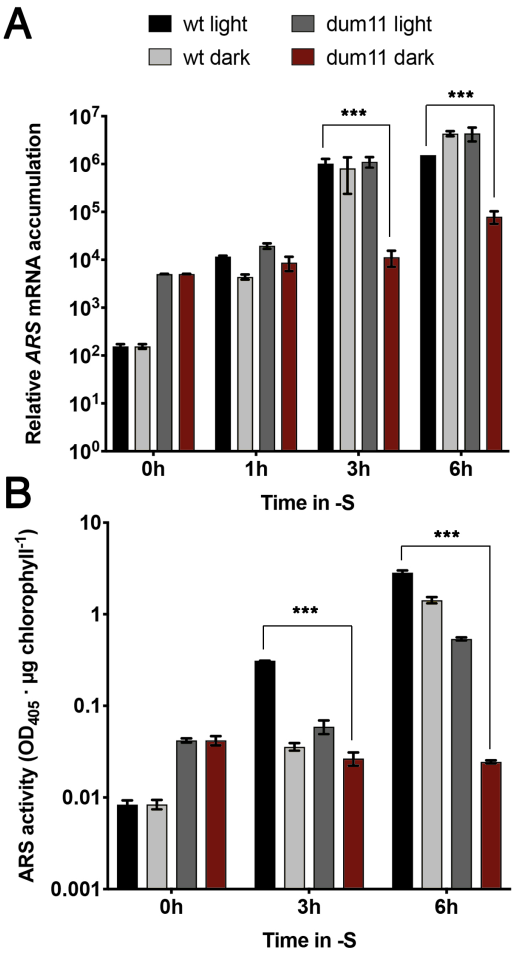

Supplementary Fig. 8. ARS activity and mRNA levels in the complex III mutant *dum11* upon S deprivation. (**A**), *ARS* mRNA accumulation and (**B**), ARS activity in S-deprived cells in the light and in the dark. In wt cells in the light both respiration and photosynthesis are active, while cells in the dark only maintain respiratory activity. In *dum11* in the light only photosynthesis is fully active, while in the dark both respiration and photosynthesis are inhibited. Error bars represent ±SD n≥3. Student's t-test was performed, p value <0.001 (***).

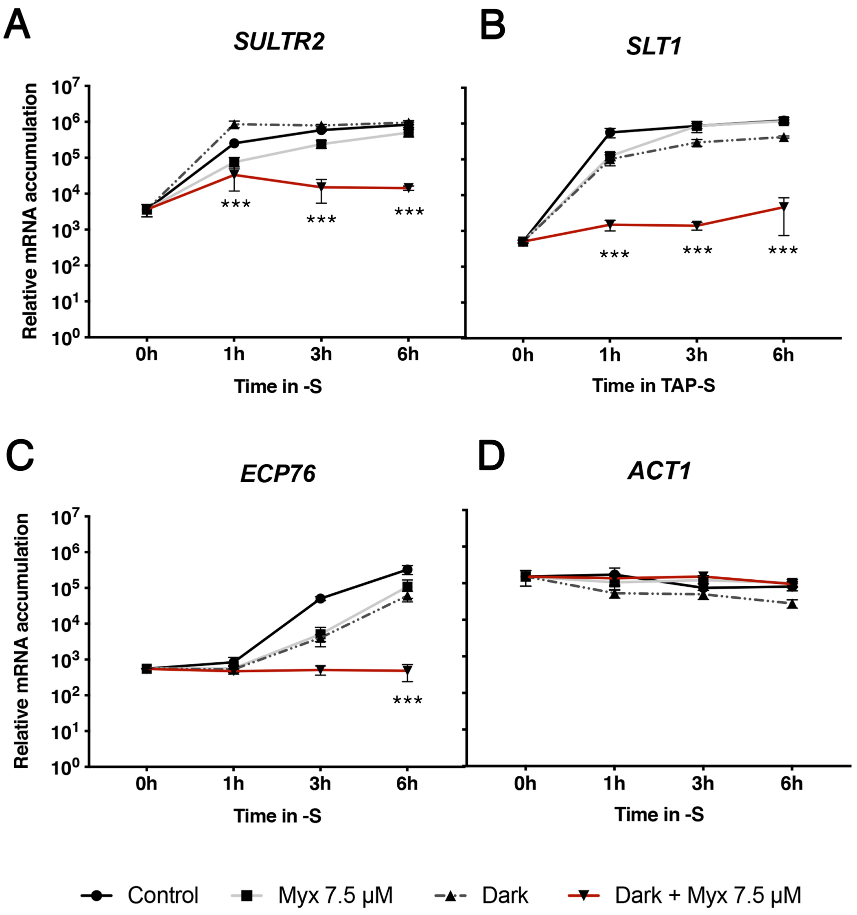

Supplementary Fig. 9. Electron flow in mitochondria or chloroplasts is required to induce *SULTR2*, *SLT1* and *ECP76*. Cells were induced in TAP-S and treated as indicated. Ethanol was used to dissolve myxothiazol (Myx) and was added to the untreated control samples. To inhibit photosynthetic electron flow, cells were incubated in the dark. Changes in (**A**), *ECP76*, (**B**), *SULTR2*, (**C**), *SLT1* and (**D**), *ACT1* mRNA accumulation were quantified at the indicated times.

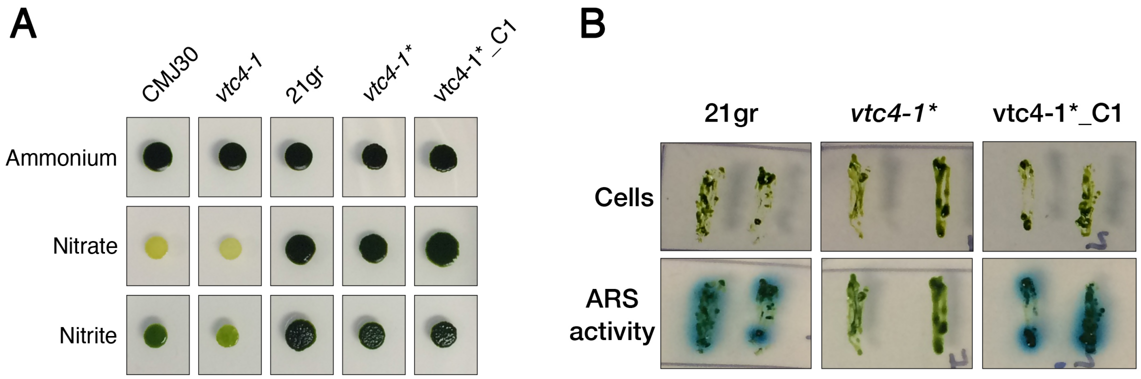

Supplementary Fig. 10. Introduction of *vtc4-1* lesion into wt 21gr. *vtc4-1* (paromomycin resistant, unable to grow with nitrate as a sole N source) was crossed with 21gr (paromomycin sensitive, grows on nitrate as a sole N source) and paromomycin resistant progeny able to grow on nitrate were selected. Only those with the 21gr genetic background for N assimilation and bearing the CIB cassette, which was used for the mutagenesis, were able to grow (designated *vtc4-1**). (**A**), Growth of the wt strains CMJ030 and 21gr, the mutants *vtc4-1* and *vtc4-1** and the complemented strain (*vtc4-1**_C1) in ammonium, nitrate and nitrite. The complemented strain was obtained by ectopic expression of *VTC4* that was transferred into the *vtc4-1** strain. (**B**), ARS activity of cells induced for 5 d in TAP-S medium. Photo credits for (A) and (B): Emanuel Sanz-Luque, Carnegie Institution for Science.
